# Establishment and Characterization of a Spermatogonial Stem Cell-like Line with Potential for Meiotic Differentiation in the Endangered Fish *Rhinogobio nasutus*

**DOI:** 10.3390/biology15191779

**Published:** 2026-10-07

**Authors:** Yue Wang, Yanbin Liu, Muhammad Jawad, Haijing Xu, Qinjun Wu, Lang Gui, Ni Hong, Zongqiang Lian, Mingyou Li

**Affiliations:** 1 Key Laboratory of Exploration and Utilization of Aquatic Genetic Resources, College of Fisheries and Life Science, Shanghai Ocean University, Ministry of Education, Shanghai 201306, China; d240100013@st.shou.edu.cn (Y.W.); jawadktk1293@gmail.com (M.J.); d220100004@st.shou.edu.cn (H.X.); m250100202@st.shou.edu.cn (Q.W.); lgui@shou.edu.cn (L.G.); nhong@shou.edu.cn (N.H.); 2 Ningxia Fisheries Research Institute, Yinchuan 750001, China; yb_liu8208@163.com; 3 Ningxia Fishery Engineering Technology Research Center, Yinchuan 750001, China

**Keywords:** *Rhinogobio nasutus*, spermatogonial stem cells, cell transplantation, sperm-like morphology, germplasm conservation

## Abstract

*Rhinogobio nasutus*, a freshwater fish belonging to the family Cyprinidae, is primarily distributed in the upper and middle reaches of the Yellow River. However, overfishing and habitat degradation have resulted in a marked decline in its wild populations, and the species was listed as a Class II nationally protected aquatic wildlife species in China in 2021. Spermatogonial stem cells (SSCs) provide a valuable resource for fish germplasm conservation and reproductive biotechnology. In this study, we successfully established a long-term cultured germ-cell population with spermatogonial stem cell-like characteristics derived from *R. nasutus* (RnSSCs). These cells exhibited a morphology consistent with an undifferentiated spermatogonial phenotype and showed a diploid chromosome number of 2*n* = 50. Alkaline phosphatase (AP) staining showed strong positive activity, while RT-PCR detected the expression of germ-cell- and stem-cell-associated markers, with only weak expression of the somatic-cell-associated marker. Immunofluorescence staining revealed strong signals for both proliferating cell nuclear antigen (PCNA) and the germ cell-specific protein Vasa. After more than four months of *in vitro* culture, the cells continued to proliferate and retained expression of germ-cell- and stem-cell-associated markers. Following transplantation into zebrafish blastulae, fluorescently labelled cells were detected in the gonadal region of a small number of recipient embryos. Following hormonal induction, some cells developed a sperm-like morphology, accompanied by the expression of the meiotic-associated markers *rec8* and *dmc1*. The establishment of the RnSSC line provides a renewable cellular resource for studying spermatogenesis and germ cell transplantation in *R. nasutus.* These findings provide a useful cellular resource for studying the reproductive biology of *R. nasutus*.

## Simple Summary

*Rhinogobio nasutus* is a freshwater fish species primarily distributed in the upper and middle reaches of the Yellow River. However, overfishing and the severe degradation of its natural habitat have led to a marked decline in its population, threatening the conservation of its genetic resources. To support future conservation and reproductive studies of this species, we report the first successful establishment of a long-term cultured germ-cell population with characteristics of spermatogonial stem cell-like line derived from *R. nasutus* (RnSSC). The cultured cells exhibited a morphology consistent with an undifferentiated spermatogonial phenotype and showed a diploid chromosome number of 2*n* = 50, as well as sustained expression of germ-cell and stem-cell-associated markers during more than four months of *in vitro* culture. Transplantation experiments showed that labelled cultured cells survived in recipient zebrafish embryos, with a small number of cells detected in the gonadal region. Furthermore, following hormonal induction, some cells developed a sperm-like morphology, accompanied by the expression of meiosis-associated markers. These findings provide a useful cellular resource for studying the reproductive biology of *R. nasutus*.

## 1. Introduction

Fish represent the most diverse group of vertebrates and serve as a critical source of dietary protein worldwide [1,2]. However, anthropogenic pressures such as over-exploitation, habitat destruction, and water pollution have led to substantial declines in fish biodiversity, placing many species at risk of extinction [1]. Consequently, effective strategies for long-term conservation of fish germplasm are urgently needed, particularly for threatened and endangered species. In recent years, advances in reproductive biotechnology have highlighted the potential of culturing and cryopreserving fish germline stem cells (GSCs) as a promising approach to preserving genetic diversity [3,4]. Unlike the cryopreservation techniques for sperm, embryos, or intact gonadal tissues, the preservation of fish GSCs not only enables continuous *in vitro* expansion but also allows long-term storage and highly efficient proliferation after revival. Fish germline stem and progenitor cells encompass populations at different developmental stages, including primordial germ cells (PGCs), spermatogonial stem cells (SSCs), and oogonial stem cells (OSCs). Among these, SSCs have been studied most extensively and have comparatively well-established in vitro culture systems.

SSCs are adult stem cells residing in the testis that possess the unique ability to self-renew and differentiate into functional spermatozoa, thereby transmitting genetic information across generations [5,6,7]. Therefore, long-term *in vitro* culture of SSCs provides an important platform for investigating the mechanisms underlying their self-renewal and differentiation and for advancing reproductive biotechnology in aquaculture. In 2004, the first long-term cultured fish SSC line capable of producing sperm was established from the adult testis of the model species medaka (*Oryzias latipes*) [8]. Subsequently, SSC culture systems were investigated in several other fish species, although long-term proliferation has remained difficult to achieve in most of them. In 2022, an *in vitro* SSC culture from the economically important Chinese hook snout carp (*Opsariichthys bidens*) was established using the SSC culture protocol developed by Hong et al. [9]. Following this achievement, SSCs from *Coilia nasus*, grouper (*Epinephelus coioides*), and pufferfish (*Takifugu rubripes*) were successively cultured *in vitro* [10,11,12]. Nevertheless, successful *in vitro* propagation remains challenging in certain species such as rainbow trout, common carp, and eel, in which cultured spermatogonial cells have been reported to sustain proliferation for only short periods (<1 month), indicating significant interspecies variability in culture requirements [13,14,15,16]. Moreover, while progress has been made in model or economically important fish species, the establishment of stable SSC lines from large-bodied, slow-maturing, or endangered fish species remains challenging because of limited biological material and the lack of species-specific culture protocols.

With the continuous development of *in vitro* SSC culture techniques, refinement of SSC culture conditions through the supplementation of growth factors and reproductive hormones has enabled the induction of spermatogenic differentiation in vitro by partially recreating the gonadal microenvironment [17]. In mammals, mouse pluripotent stem cells completed meiosis and generated haploid male germ cells under three-dimensional (3D) culture systems [18,19]. In fish, various *in vitro* culture systems have successfully achieved the differentiation of SSCs into sperm-like cells. For example, when medaka SG3 cells were co-cultured with rainbow trout stromal cells, morphological, gene expression, and morphological analyses revealed that SG3 retained the potential to undergo meiosis and form normal sperm *in vitro* [8]. Similarly, cytomorphological and DNA content analysis of Chinese hook snout carp SSCs in co-culture with testicular somatic cells demonstrated that these SSCs retain meiotic competence and maintain spermatogenic potential. [9]. After 12 days of co-culturing *Coilia nasus* SSCs with somatic cells, elongated spermatid-like with slender tails were morphologically observed, accompanied by the expression of meiotic-related genes [10]. These findings provided evidence of meiotic-associated differentiation and the formation of sperm-like cells in cultured *Coilia nasus* cells. Additionally, treatment with 11-ketotestosterone induced the differentiation of grouper SSCs into sperm-like cells, which were further characterized by cytomorphology and gene expression patterns [11]. In the four-eyed sleeper (*Bostrychus sinensis*), researchers applied composite hormone treatment to freshly isolated spermatogonia for one month, successfully cultivating motile sperm with flagella and producing viable offspring [20]. In summary, current studies on long-term *in vitro* culture and induction of SSCs primarily focus on methodological exploration. The identification of derived sperm-like cells mainly relies on cytomorphological observation and gene expression analysis. Although some studies have reported flagellated or functionally competent sperm following in vitro differentiation, successful production of viable offspring from long-term cultured fish SSC lines remains rare.

*Rhinogobio nasutus* is a cyprinid freshwater fish belonging to the subfamily Gobioninae and is primarily distributed in the upper and middle reaches of the Yellow River [21]. Wild populations of this species have declined markedly because of habitat degradation and intensive fishing pressure. In 2007, the Chinese Ministry of Agriculture designated the Qingshi section of the Yellow River in Ningxia as a national aquatic germplasm resource reserve for *R. nasutus* [22]. In 2021, the species was listed as a Class II nationally protected aquatic wildlife species in China [23]. To date, research on *R. nasutus* remains limited, with studies mainly addressing muscle nutritional composition, mitochondrial genetic diversity, and genomic characteristics [24,25,26]. Information on its reproductive biology is particularly scarce. Previous studies have described embryonic development and larval morphology [27], while more recent transcriptomic analyses have provided molecular insights into sex differentiation through integrated mRNA and miRNA profiling [28]. Because artificial breeding protocols for *R. nasutus* have not yet been established, coupled with its lengthy reproductive cycle (reaching sexual maturity at 3 years), the necessity of adopting alternative methods to protect and utilize its germplasm resources becomes evident. Compared with traditional conservation approaches such as establishing nature reserves, stock enhancement or ex situ conservation, SSC culture is not restricted by breeding seasons, allows expansion of cultured germ cells from limited biological material, and may be combined with germ-cell differentiation technologies to support germplasm conservation in endangered species. To date, there has been no established SSC culture system for *R. nasutus*. Therefore, establishing and characterizing such a culture is the specific knowledge gap addressed here. In the present study, we established a long-term cultured germ-cell population with characteristics of spermatogonial stem cell-like line derived from *R. nasutus* (RnSSC). The germ-cell- and stem-cell-associated characteristics were evaluated through molecular biological methods. Following transplantation into zebrafish embryos, PKH26-labelled fluorescent cells were detected in the gonadal region of recipient zebrafish. Additionally, we investigated whether hormonal treatment was associated with morphological differentiation and activation of meiosis-associated genes in the cultured cells. The establishment and characterization of this cultured germ-cell population provide a cellular resource for investigating the reproductive biology of *R. nasutus* and may support future studies of germplasm conservation and reproductive biotechnology.

## 2. Materials and Methods

### 2.1. Ethical Statement

All experimental procedures involving *R. nasutus* were approved by the Animal Care and Use Committee of Shanghai Ocean University (SHOU-2023-031 on 1 September 2023).

### 2.2. Experimental Fish and Culture Medium Preparation

Seven-month-old male *R. nasutus* were obtained from the Ningxia Institute of Fisheries Science (Yinchuan, Ningxia, China). These fish were the artificially bred offspring of wild parental stock originally sourced from the Yellow River. Fish were reared in 500-L circular tanks at 25 °C with a photoperiod of 14 h light/10 h dark and were fed commercial pellets twice daily. The culture medium was modified from the formulation described by Yi et al. [29]. Briefly, DMEM containing HEPES buffer (pH = 7.8; Gibco, New York, NY, USA) was supplemented per liter with: fetal bovine serum (FBS, 150 mL; Gibco, Macquarie Park, NSW, Australia), non-essential amino acids, sodium pyruvate (1 mM), L-glutamine (2 mM), 100 IU/mL penicillin, 0.1 mg/mL streptomycin (all 100×; 10 mL; Gibco, New York, NY, USA), sodium selenite (1 mL; stock 2 μM; Sigma, Burlington, MA, USA), β-mercaptoethanol (2 mL; stock 50 mM; Sigma), basic fibroblast growth factor (bFGF, 10 ng/mL; Gibco, Carlsbad, CA, USA), *R. nasutus* serum (2 mL; prepared as previously described [29]), and medaka embryo extract (1 mL, equivalent to 1000 embryos/mL; prepared as previously described [29]). The complete culture medium was filtered through a 0.22-μm membrane filter and stored according to the Yi et al. protocol [29].

### 2.3. Primary Cell Culture and Subculture

All instruments were sterilized prior to use. Three seven-month-old males (each fish weighs approximately 10–15 mg) were placed on ice for 10 min and the body surface was disinfected with 75% ethanol. Testes were excised and rinsed three times in phosphate-buffered saline (PBS) containing 1% penicillin-streptomycin, respectively. Tissues were minced with scissors, followed by the addition of 200 μL of 0.05% trypsin (Gibco, New York, NY, USA) for digestion at 25 °C for 30 min. During this process, the mixture was gently pipetted every 10 min to ensure uniform dissociation. Digestion was terminated by adding serum-containing culture medium. Dissociated cells were separately seeded into gelatin-coated 96-well plates and incubated at 25 °C (approximately 4 × 10^4^ cells per well). Half of the culture medium was replaced every 2 days, which was used to reduce cellular debris and non-adherent cells. When confluence reached ~90%, cells were passaged at a 1:2 ratio using 0.05% trypsin. After 20 passages, a long-term cultured germ cell population with spermatogonial stem cell-like characteristics was designated RnSSC (one independent line).

### 2.4. Cryopreservation and Thawing

When RnSSCs reached approximately 90% confluence, they were harvested by digestion with 0.05% trypsin. The dissociated cells (approximately 1 × 10^6^ cells) were then resuspended in 1.5 mL freezing medium (composed of 70% culture medium, 20% FBS, 10% dimethyl sulfoxide (DMSO)) and transferred to 2 mL cryovials (Corning, Reynosa, Mexico). The cryovials were initially placed at −80 °C overnight before final transfer to liquid nitrogen for long-term storage. For the thawing process, frozen vials were rapidly thawed in a 37 °C water bath. The cell suspension was centrifuged at 400× *g* for 5 min, the supernatant was carefully removed, and the cells were resuspended in fresh culture medium. The cells were then seeded onto gelatin-coated culture dishes, fresh culture medium was added after 24 h of incubation.

### 2.5. Alkaline Phosphatase (AP) Staining

RnSSCs at passage 13 (cultured for 80 days after cryopreservation and recovery) were seeded into three 24-well plates. When the cells reached approximately 60% confluence, they were fixed with 4% paraformaldehyde for 15 min at room temperature, followed by three washes with phosphate-buffered saline (PBS). AP activity was detected using a BCIP/NBT kit (Invitrogen, Waltham, MA, USA) in accordance with the manufacturer’s instructions. The staining reaction was terminated with distilled water. AP-positive cells, exhibiting characteristic purple/blue staining, were then visualized and documented under a microscope. We randomly selected 3–5 non-overlapping fields of view to count positive cells.

### 2.6. Plasmid Transfection

RnSSCs at passage 20 (cultured for ~100 days after cryopreservation and recovery) were seeded in gelatin-coated 24-well plates. At ~70% confluence (~1 × 10^5^ cells), 1 μg of pCVpr plasmid encoding red fluorescent protein (RFP) [30] was transfected using the Neon™ NxT Electroporation System (Thermo Fisher Scientific, Waltham, MA, USA, Cat. No. N1096) (1400 V voltage, 20 ms width, 2 pulses). The cells were incubated at 25 °C for 48 h, RFP expression was examined 48 h later by fluorescence microscopy and transfection efficiency was calculated via ImageJ software (version 1.53) (randomly selected five microscopic fields from both bright-field and fluorescent-field, and Transfection rate (%) = (Number of fluorescent cells/Total cell count) × 100).

### 2.7. Chromosome Analysis

RnSSCs at passage 15 (cultured for ~90 days after cryopreservation and recovery) were seeded in 6-well plates at a density of approximately 4 × 10^5^ cells per well and maintained at 25 °C for 24 h. To induce metaphase arrest, colchicine was added to achieve a final concentration of 0.2 μg/mL, followed by a 4 h incubation period. Following incubation, cells were carefully harvested and washed twice with PBS. The cell pellet was then subjected to hypotonic treatment using 75 mM KCl solution for 30 min at room temperature to achieve proper chromosome spreading. The hypotonic treatment was terminated by adding 1 mL of freshly prepared methanol/acetic acid fixative (3:1, *v*/*v*). After 1 h of fixation at room temperature with occasional gentle mixing, the cells were centrifuged and resuspended in 300 µL of fresh fixative. Using the conventional drop technique, cell suspensions were dropped from a height of approximately 30 cm onto pre-chilled, moistened microscope slides. The slides were air-dried, stained with 5% Giemsa solution at room temperature for 15 min, and gently rinsed with distilled water. Chromosomes were observed and photographed using a microscope equipped with a 100× oil-immersion objective. At least 10 well-spread metaphases from three independent preparations were analyzed.

### 2.8. Gene Expression Analysis

Total RNA was isolated from RnSSCs, testis, and ovary tissues using Trizol reagent (Sigma, Burlington, MA, USA) following the manufacturer’s protocol. Following chloroform extraction, isopropanol precipitation, and 75% ethanol wash, RNA was dissolved in 20 μL DEPC-treated water. RNA concentration and quality were assessed by spectrophotometry and agarose gel electrophoresis. cDNA was synthesized from 1 μg of total RNA using a reverse transcription kit (ABclonal, Wuhan, China). RT-PCR amplification was performed with gene-specific primers (Table 1) under the following cycling conditions: 95 °C for 5 min; 36 cycles of 95 °C for 15 s, 60 °C for 15 s, 72 °C for 45 s; final extension at 72 °C for 7 min. β-actin served as the internal control. Amplified products were resolved on agarose gels.

### 2.9. Immunofluorescence Staining

Prior to cell seeding, 24-well cell culture plates containing glass coverslips were sterilized via overnight immersion in 75% ethanol, followed by three washes with PBS to remove residual alcohol. The plates were then coated with 0.1% gelatin and incubated at room temperature for 2 h to enhance cell adhesion. After aspirating the gelatin solution, the plates were air-dried in a sterile biosafety cabinet to prevent contamination. RnSSCs (cultured for ~90 days after cryopreservation and recovery) were seeded onto the coated plates and cultured until reaching ~70% confluence. Cells were then gently fixed in 4% paraformaldehyde for 10 min, ensuring optimal preservation of cellular architecture. After fixation, cells were permeabilized with 0.3% Triton X-100 for 10 min to facilitate antibody penetration, followed by blocking with 4% BSA for 30 min to minimize nonspecific binding. Then, samples were incubated overnight at 4 °C with primary antibodies: Vasa antibody (Rabbit-derived antibody; 1:200 dilution with 4% BSA; Invitrogen, Waltham, MA, USA, PA5-30749) and proliferating cell nuclear antigen (PCNA) antibody (Mouse-derived antibody; 1:200 dilution with 4% BSA; Sigma, P8825). After three PBS washes, secondary antibodies—goat anti-rabbit FITC (1:100 dilution with 4% BSA; Invitrogen, A-11034) and goat anti-mouse TRITC (1:100 dilution with 4% BSA; Invitrogen, A-11029)—were applied for 1 h at room temperature in the dark. Following secondary antibody incubation, cells were washed three times with PBS and stained with DAPI (1:500 dilution with PBS, Sigma, D9542) for 5 min to visualize nuclei. Coverslips were mounted using an antifade mounting medium (Invitrogen, Carlsbad, CA, USA) to prevent fluorescence quenching. Images were observed under Nikon inverted fluorescence microscope.

### 2.10. Cell Transplantation

Cell transplantation was performed as previously described [31,32]. Briefly, RnSSCs (cultured for ~120 days after cryopreservation and recovery) were cultured on gelatin-coated 12-well plates until reaching 80% confluency. For cell tracking, cultures were labeled with PKH26 red fluorescent dye according to the manufacturer’s protocol (Sigma-Aldrich, St. Louis, Mo, USA). Labeled cells were gently harvested and maintained on ice for immediate use. Sixty dechorionated wild zebrafish embryos came from one experiment were positioned under a microinjection system. At the mid-blastula stage (~4 h post-fertilization), approximately 50–100 RnSSCs were transplanted into the deep cell layer of the animal pole using a calibrated glass capillary (inner tip diameter: ~30 μm). Injected embryos were placed in 10 cm dishes (divided into three groups with 20 embryos per group) containing sterile embryo culture medium (per 1 L: 11 g NaCl, 0.8 g KCl, 0.4 g MgSO_4_·7H_2_O, 0.4 g CaCl_2_·2H_2_O, 10 g PEG 2000, 1 mg phenol red, 10 mL 100× penicillin/streptomycin, 5 mL 5% NaHCO_3_, the final volume was adjusted to 1 L with double distilled water) and maintained at 28 °C with daily medium changes.

### 2.11. Hormonal Induction and Analysis of Meiosis-Associated Markers Expression

To investigate hormone-associated differentiation of the cultured germ cell population, induction medium was prepared according to Zhang et al. [20] with minor modifications. Briefly, the induction medium consisted of standard stem cell culture medium enriched with 10 IU/mL human chorionic gonadotropin (hCG), 200 ng/mL 11-ketotestosterone (Acmec, Shanghai, China), 50 ng/mL 17α,20β-dihydroxy-4-pregnen-3-one (Cayman, Wuhan, China), and 10 U/mL pregnant mare serum gonadotropin (PMSG; Solarbio, Beijing, China). Control cultures received stem cell culture medium without hormones. In six 12-well plates, approximately 9 × 10^4^ cells were seeded in each well. Three plates served as the control group, while the other three constituted the hormone-induced group. Both groups of cells were cultured under their respective conditions, with half of the culture medium being replaced every three days. Under microscopic observation, non-overlapping fields of view were randomly selected to calculate the proportion of differentiated cells. On day 16, cells from induced and control groups were harvested for molecular analysis. RT-PCR was performed to evaluate the expression of the meiosis-associated markers *rec8* and *dmc1* using primers detailed in Table 1.

## 3. Results

### 3.1. Establishment, Cryopreservation and Plasmid Transfection of the Rhinogobio nasutus Spermatogonial Stem Cell-like Cells Line

**Figure 1:**
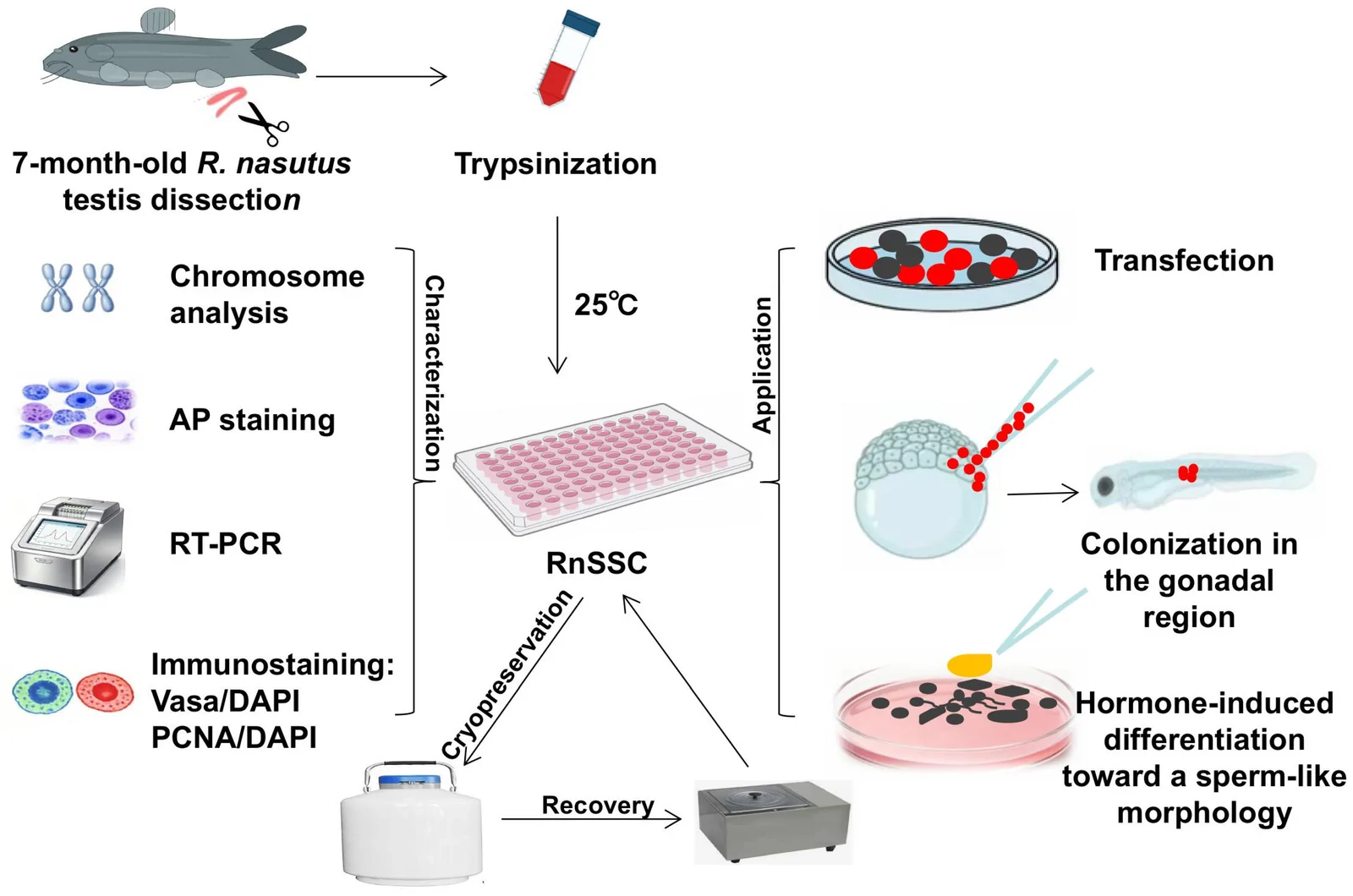
Experimental workflow. This study utilized a 7-month-old male *R. nasutus*. The live fish was euthanized on ice, and testicular tissue was aseptically dissected using sterile scissors. The tissue was rinsed three times with PBS containing 1% penicillin-streptomycin, mechanically minced, and enzymatically digested with trypsin to obtain a single-cell suspension for primary culture. Following serial passaging under optimized conditions, a stable spermatogonial stem cell-like characteristics derived from *R. nasutus* (RnSSCs) was established. The RnSSC-like cells line was characterized via alkaline phosphatase (AP) staining, karyotyping, RT-PCR, and immunofluorescence analysis. Validated cell line was cryopreserved in liquid nitrogen for archival storage. Notably, electroporation-mediated exogenous gene delivery was successfully performed in RnSSCs. In functional assays, PKH26-labeled cells were detected in the gonadal region of a small subset of recipient zebrafish embryos. Following hormonal induction, some cells developed a sperm-like morphology accompanied by the expression of the meiotic-associated markers *rec8* and *dmc1*.

**Figure 2:**
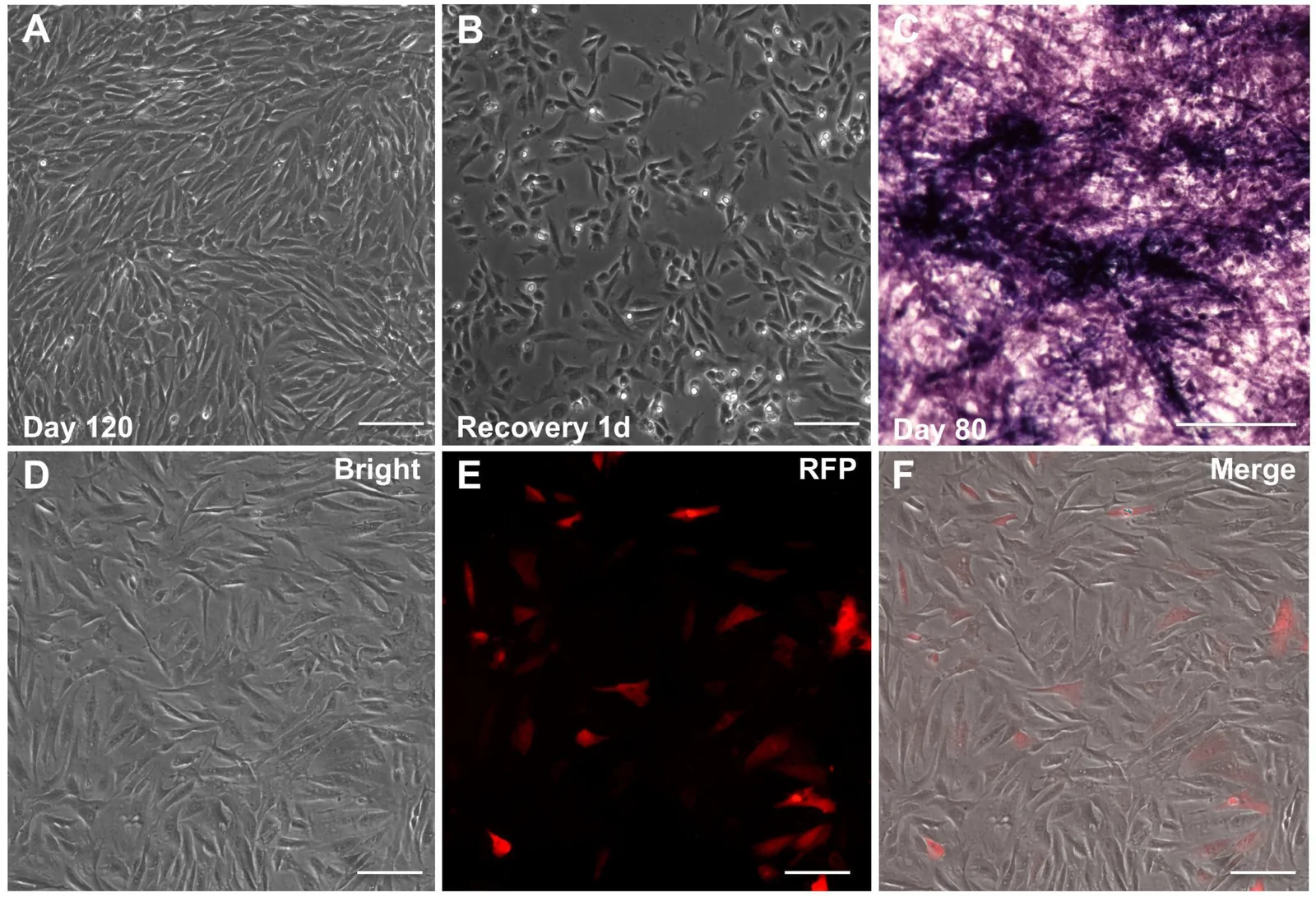
Primary culture, cryopreservation, alkaline phosphatase staining and transfection of RnSSC-like cells. (**A**) *In vitro* culture of RnSSC-like cells for 120 days. (**B**) Recovery status of RnSSC-like cell after 75-day culture followed by cryopreservation. (**C**) Alkaline phosphatase staining performed on RnSSC-like cells at 80 days post-thaw cultivation. (**D**–**F**) Bright (**D**), Fluorescence (**E**), and Merge (**F**) images of RnSSC-like cells 48 h after electroporation with the pCVpr plasmid. (Bars = 100 µm).

The establishment process of the *Rhinogobio nasutus* spermatogonial stem cell-like line (RnSSC) is illustrated in Figure 1. Testicular tissues obtained from 7-month-old male *R. nasutus* were subjected to enzymatic digestion using 0.05% trypsin to generate a single-cell suspension. The resulting single-cell suspension was seeded into gelatin-coated 96-well plates and cultured at 25 °C. Approximately 50% of the cells successfully adhered by day 7 post-plating. The cells reached full confluence in the culture plate by day 15 and were subsequently passaged at a 1:2 split ratio (Figure S1A–E). Notably, the proliferative capacity of the cultured cells exhibited progressive enhancement with each subsequent passage, indicating proliferative capacity increased during serial passaging. After more than four months of continuous culture, RnSSC-like cells maintained stable proliferation (Figure 2A) and retained morphological features consistent with an undifferentiated spermatogonial phenotype, including small cell size, distinct cytoplasmic boundaries, sparse cytoplasm, and prominent nucleoli (Figure S1F). The cryopreserved RnSSC-like cells cultured *in vitro* for 75 days were thawed and subsequently seeded onto gelatin-coated 6-well plates. Within 24 h, approximately 60% of the cells reattached to the culture surface (Figure 2B), and the cells subsequently resumed proliferation, reaching approximately 90% confluence by day 3. These observations showed that RnSSC-like cells retained proliferative capacity after cryopreservation and recovery.

To evaluate the stemness properties of the established RnSSC-like cells, AP staining was conducted following 80 days of post-thaw cultivation. The cultured cells exhibited strong AP-positive staining (Figure 2C), indicating retention of an undifferentiated stem-cell phenotype during prolonged culture.

To assess exogenous gene introduction into the established cell line, RnSSC-like cells were electroporated with the RFP-expressing pCVpr plasmid. At 48 h post-transfection, approximately 30% of the cells exhibited red fluorescence (Figure 2D–F). These results demonstrate the feasibility of transient plasmid-mediated fluorescent labeling of the cultured cells.

### 3.2. Characterization of RnSSC-like Cells

**Figure 3:**
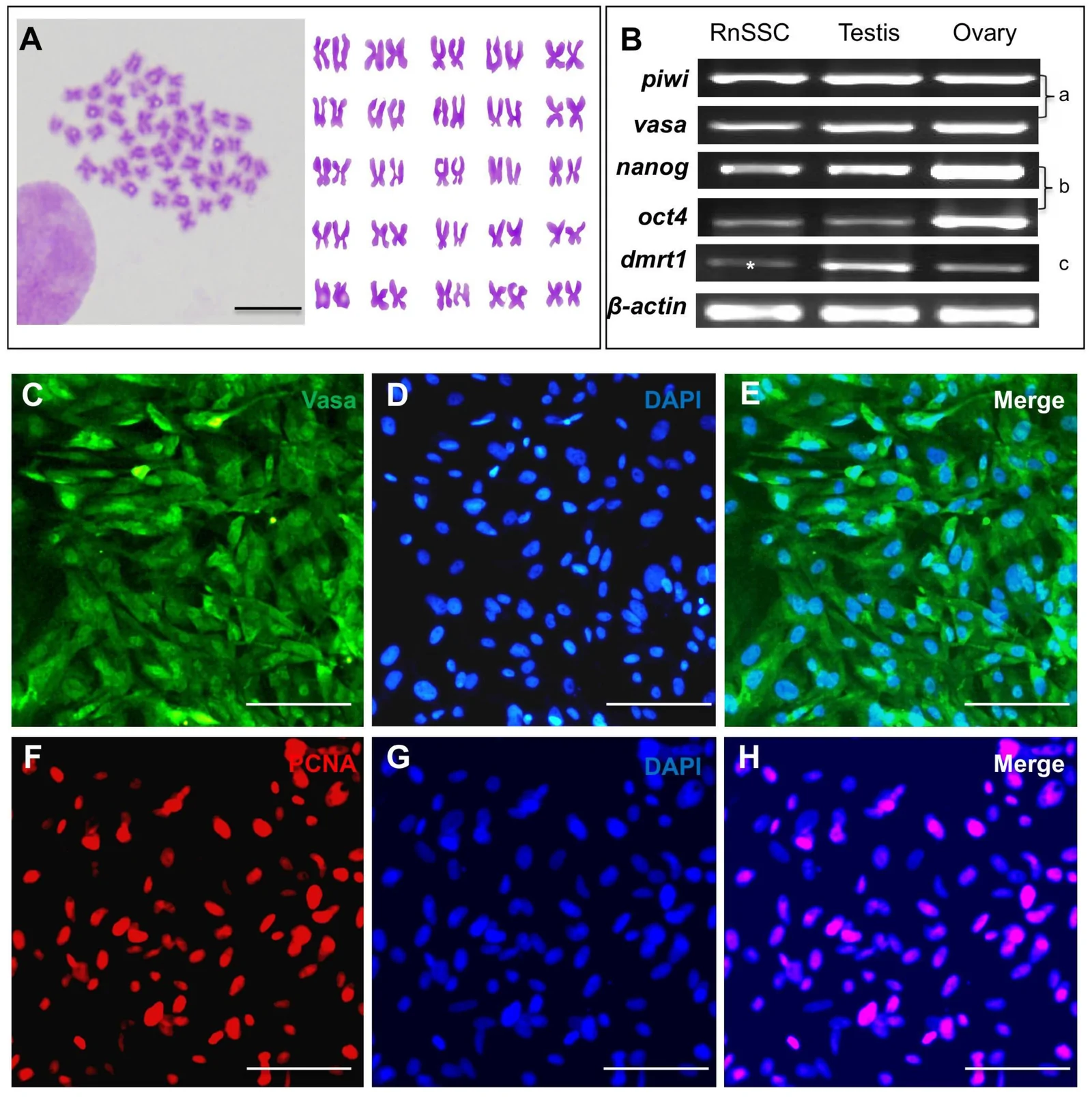
Chromosome analysis, RT-PCR, and immunofluorescence analysis of RnSSC-like cells. (**A**) Chromosomal morphology of RnSSC-like cells at passage 15 (2n = 50) (Bar = 10 µm). (**B**) RT-PCR analysis of germ cell markers (a), stem cell markers (b), and somatic cell marker (c) in RnSSC-like cells and gonadal tissues. * represents *dmrt1* expression in RnSSC-like cells. (**C**–**H**) Immunofluorescence staining of Vasa (green) and PCNA (red) in RnSSC-like cells. (Bars = 100 µm).

Chromosome analysis identified cells with a diploid chromosome number of 2*n* = 50 (Figure 3A), indicating maintenance of the normal chromosome number during prolonged in vitro culture. These observations indicate that cells with the expected diploid chromosome number were present after prolonged culture. To further characterize the identity of the cultured cells, we examined the expression of germ-cell-associated, stem-cell-associated, and somatic-cell-associated markers by RT-PCR. RnSSC-like cells expressed the germ-cell-associated markers *piwi* and *vasa* and the stem-cell-associated markers *nanog* and *oct4*, whereas the gonadal somatic-cell-associated marker *dmrt1* was only weakly expressed (Figure 3B, refer to the original image in Figure S2). Immunofluorescence staining further revealed Vasa signals in the cytoplasm of RnSSCs (Figure 3C–E) and nuclear PCNA signals (Figure 3F–H). Together, these results indicate that the long-term cultured RnSSC-like cells retained characteristics consistent with an undifferentiated spermatogonial phenotype.

### 3.3. In Vivo Survival and Distribution of Transplanted RnSSC-like Cells

**Figure 4:**
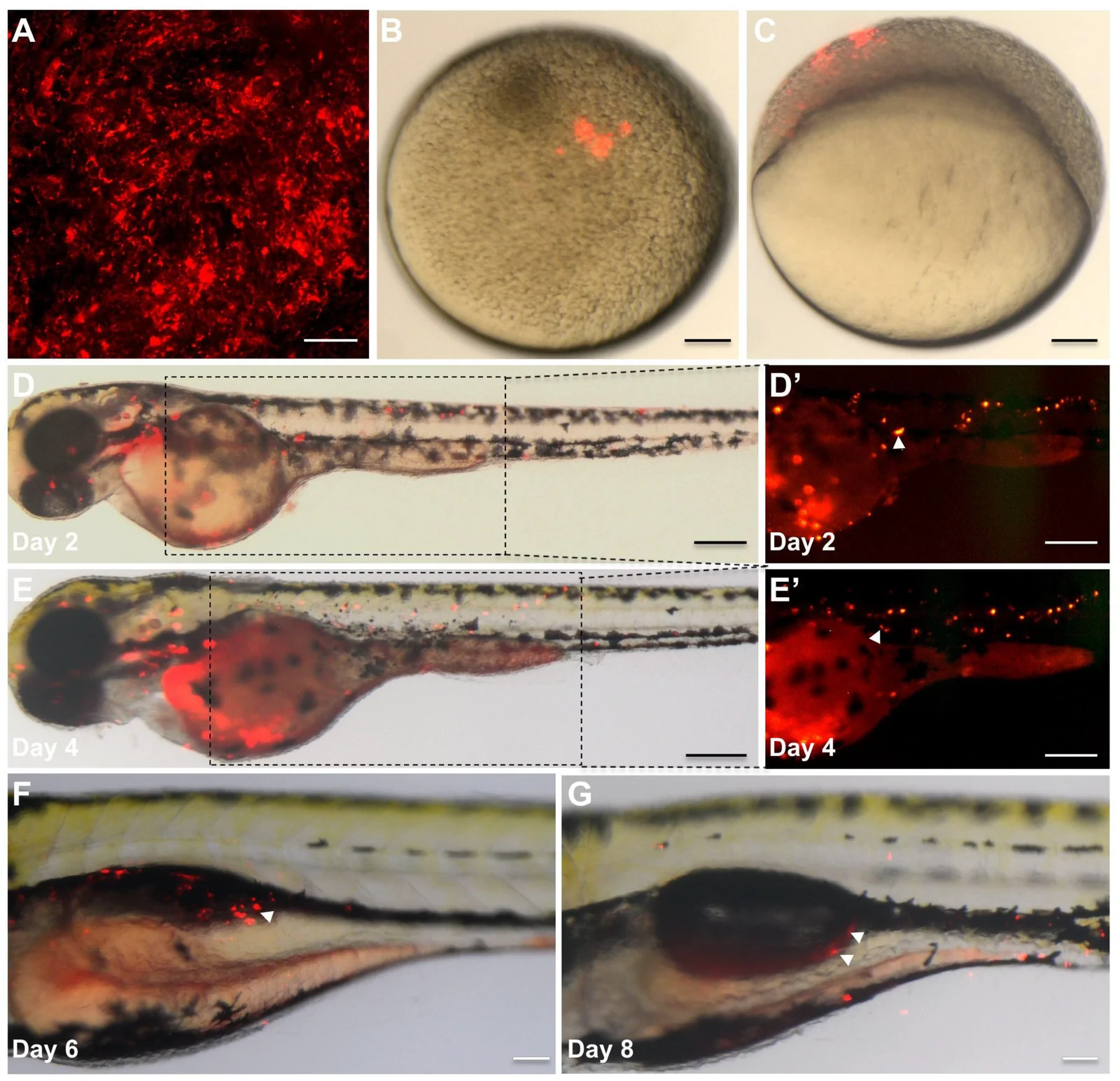
Engraftment dynamics of PKH26-labeled RnSSC-like cells in zebrafish embryos across developmental stages. (**A**) Fluorescent labeling of RnSSC-like cells with PKH26. (**B**–**G**) Detection and distribution of PKH26-labelled cells in zebrafish embryos across developmental stages Overlay images of bright-field and fluorescence channels were shown in (**B**–**G**). White triangles indicate PKH-26 labeled fluorescent cells migrating to recipient gonadal region. Fluorescence images of the boxed regions (dashed black lines) in (**D**,**E**) were displayed in (**D’**,**E’**). (Bars = 100 µm).

To rigorously evaluate the developmental plasticity of in vitro-maintained RnSSC-like cells, we performed a xenogeneic transplantation model using zebrafish embryos. RnSSC-like cells cultured *in vitro* for four months were labeled with PKH26 red fluorescent dye and micro-transplanted into the deep cell layer of dechorionated zebrafish embryos at the blastula stage. From 60 transplanted embryos, 37 survived (61.7% survival rate), with 30 survivors maintaining detectable PKH26-labelled fluorescent cells during the one-week observation period. Transplanted RnSSC-like cells were distributed in several regions of the developing recipients, including the brain, muscle, notochord, and epidermal tissues in 27 embryos. Notably, PKH26-labelled fluorescent cells were detected in the gonadal region of three transplanted embryos (Figure 4). Fluorescently labelled transplanted cells were detected in several regions of developing zebrafish embryos, including the gonadal region in three embryos, indicating that a subset of transplanted cells remained detectable in recipient embryos during development.

### 3.4. Hormone-Induced Differentiation Toward a Sperm-like Phenotype Accompanied by Induction of Meiotic-Associated Markers Expression

**Figure 5:**
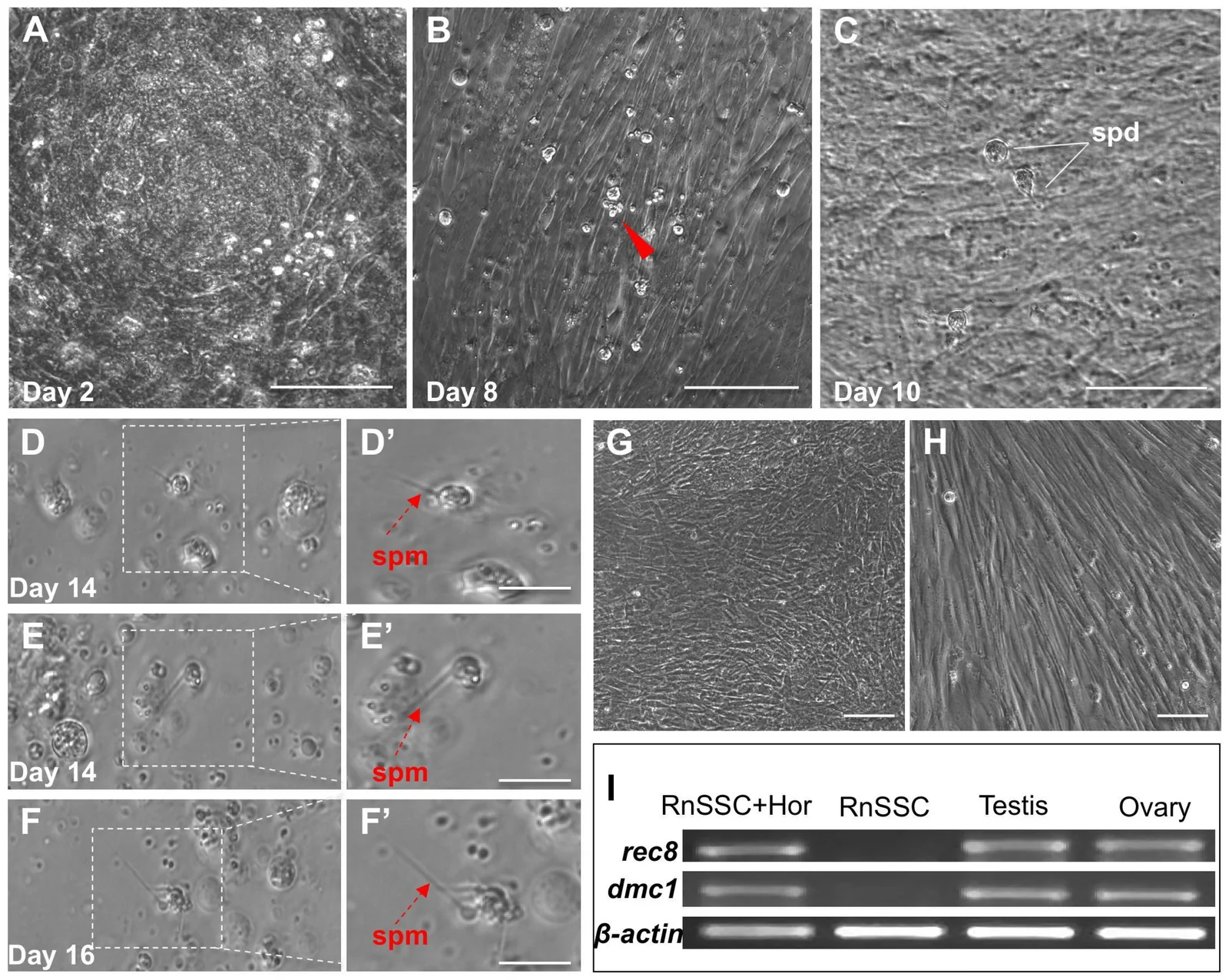
Hormone-induced differentiation toward a sperm-like phenotype accompanied by induction of meiotic-associated markers expression. (**A**) Spherical aggregates appeared at day 2 post-induction. (**B**) Four round spermatid-like cells arranged in a tetrad-like configuration (red arrow). (**C**) Round spermatid-like (spd) and elongating spermatid-like at day 10. (**D**–**F**) Cells with spermatid-like morphology subsequently developed sperm-like morphology (spm). (**D’**–**F’**) Magnified view of the region by the white dashed box in (**D**–**F**). (**G**) Control cells maintained an undifferentiated stem-cell-like morphology. (**H**) Hormone-induced cells predominantly exhibited elongated, fibroblast-like morphology. (**I**) RT-PCR detection of meiotic-associated markers transcripts (*rec8*, *dmc1*) in hormone-induced RnSSC-like cells, control RnSSC-like cells, and gonadal tissue. Both genes were expressed in the hormone-treated group, not in uninduced group. ((**A**–**C**): Bars = 100 μm; (**D’**–**F’**): Bars = 20 μm; (**G**,**H**): Bars = 100 μm).

To investigate the capacity of RnSSC-like cells for hormone-mediated differentiation into sperm-like phenotypes under *in vitro* conditions, RnSSC-like cells cultured for 120 days were transferred into gelatin-coated 12-well plates and exposed to hormone-supplemented induction medium. Compared with the control group, hormone-treated cells formed spherical aggregates by day 2 (Figure 5A), and these aggregates gradually increased in size during culture. By day 8, round spermatid-like cells arranged as tetrads were observed (Figure 5B). At day 10, a subset of spermatid-like cells exhibited an elongated morphology (Figure 5C). By day 14, cells with flagellum-like structures were observed, and by day 16, some induced cells displayed condensed heads and elongated flagella, resembling sperm-like cells (Figure 5D–F’). In addition to these germ-cell-like changes, some hormone-treated cells exhibited morphologies resembling other somatic cell types, as shown in Figure S3A–D.

In contrast, control cells continued to proliferate and formed confluent monolayers, whereas hormone-treated cultures showed marked morphological heterogeneity, including elongated fibroblast-like cells and a smaller population of sperm-like cells (Figure 5G,H). To investigate the induction of meiosis-associated markers expression, we examined *rec8* and *dmc1* by endpoint RT-PCR. As shown in Figure 5I (refer to the original image in Figure S4), both genes were detected in the hormone-treated group but were not detected in the untreated cultures, indicating that hormone treatment was associated with the induction of *rec8* and *dmc1* expression.

## 4. Discussion

In the study, we report the first successful establishment of a long-term cultured germ-cell population with SSC-like characteristics derived from *Rhinogobio nasutus*, an endangered freshwater fish species endemic to the upper and middle reaches of the Yellow River. The cultured cell population was maintained *in vitro* for more than four months without feeder layers and retained morphological, molecular, and cytogenetic characteristics consistent with an undifferentiated spermatogonial phenotype. Moreover, transplantation into zebrafish embryos showed that cultured RnSSCs survived and distributed within developing recipients, with fluorescently labelled cells detected in several embryonic regions and in the gonadal region of a small subset of recipients. These observations provide preliminary evidence that the cultured cells remained detectable following transplantation, but do not establish functional germline colonization. Furthermore, hormone treatment was associated with the appearance of a subset of cells exhibiting sperm-like morphology. RnSSCs also resumed proliferation after cryopreservation and thawing while maintaining their characteristic morphology. Collectively, these findings demonstrate that the study established a long-term cultured germ-cell population with characteristics consistent with an undifferentiated spermatogonial phenotype and demonstrated hormone-associated differentiation toward sperm-like morphology.

Constructing an optimal *in vitro* microenvironment is critical for maintaining stable proliferation of SSCs. Previous studies have demonstrated substantial species-dependent variation in the culture requirements and long-term maintenance of fish SSCs. In zebrafish, SSCs could be maintained only for a limited period under culture conditions containing fetal bovine serum (FBS) [33], while rainbow trout (*Oncorhynchus mykiss*) SSCs required insulin, trout embryo extract, and basic fibroblast growth factor (bFGF) yet showed only 1-month viability [13]. A major advance was achieved in medaka, in which Hong et al. established a long-term spermatogonial cell line using modified DMEM supplemented with amino acids, medaka embryo extract, fish serum, and growth factors [8]. Subsequent studies adapted similar culture strategies to other fish species, including Chinese hook snout carp, *Coilia nasus*, grouper, and common carp resulting in the establishment of long-term SSC cultures [9,10,11,15]. In contrast, for tiger puffer fish (*Takifugu rubripes*) SSC *in vitro* culture experiments, scientists successfully maintained puffer fish SSCs for over a year by using L15 medium as the base and supplementing with Japanese flounder embryo extract, Japanese flounder serum, basic fibroblast growth factor, and recombinant human leukemia inhibitory factor [12]. These findings demonstrate that SSC culture relies heavily on embryo extracts, serum, and growth factors, which likely provide essential components for sustaining their growth and proliferation *in vitro*. In the present study, application of the medaka-derived culture system containing perch serum did not support sustained proliferation of *R. nasutus* testicular cells. In contrast, replacement of perch serum with *R. nasutus* serum markedly improved cell attachment and proliferation. So, the final culture system incorporated *R. nasutus* serum. One possible explanation is that the observed difference reflects species-specific nutritional or growth-factor requirements rather than phylogenetic distance alone. However, this hypothesis requires further experimental validation. The resulting culture system maintained RnSSCs with stable morphology and proliferative capacity, and provided sufficient cell resources for subsequent transplantation and hormone-induced spermatogenic differentiation experiments. These findings emphasize the importance of selecting appropriate serum and other culture components when establishing long-term germ-cell cultures from previously uncharacterized fish species.

Identification of putative germline stem cells commonly relies on the combined assessment of germ-cell and stem-cell marker expression [34]. *Vasa* is a widely conserved germ-cell-associated markers in fish and is expressed in spermatogenic cells of zebrafish, tilapia, and rainbow trout [35,36,37]. PIWI-family proteins are also closely associated with germ-cell development and have important roles in the maintenance and development of the germline [38,39]. The stem-cell-associated factors *nanog* and *oct4* have been implicated in maintaining an undifferentiated state in fish stem and germ cells, whereas *dmrt1* is predominantly associated with testicular somatic cells in several fish species [40,41,42]. In this study, the germ-cell-associated markers *piwi* and *vasa*, along with the stem-cell-associated markers *nanog* and *oct4*, were co-expressed in RnSSC. This expression profile supports the predominantly germ/stem-cell character of the established culture. Furthermore, alkaline phosphatase (AP) activity, which is commonly associated with an undifferentiated stem-cell state [43], remained strongly positive in RnSSCs after 80 days of culture. Similar AP-positive phenotypes have been reported in long-term cultured SSCs from Chinese hook snout carp, grouper, and tiger puffer fish [9,11,12]. However, similar phenotypes do not establish equivalence of the cell populations. Together with their sustained proliferation, diploid karyotype, and Vasa/PCNA immunoreactivity, these molecular and cytological characteristics support the successful establishment of a long-term cultured germ-cell population with characteristics of spermatogonial stem cell-like line derived from *R. nasutus*. However, the expression of a few marker genes alone is insufficient to confirm SSC identity. While RT-PCR serves as a molecular reference, definitive assessment of SSC function requires functional assays demonstrating germline contribution, such as donor-derived spermatogenesis and, where feasible, transmission of donor genetic material to offspring.

The conservation status of *Rhinogobio nasutus*, together with its prolonged reproductive cycle, highlights the need for alternative approaches to preserve its germplasm resources. Interspecific germ cell transplantation, a key component of surrogate reproduction, has emerged as a promising strategy for the conservation and propagation of valuable fish germplasm [44]. In the present study, RnSSCs maintained *in vitro* for four months were successfully transplanted into zebrafish blastulae. During the one-week observation period, 37 out of 60 transplanted embryos survived, among which 30 exhibited PKH26-labelled fluorescent cells. Notably, only 3 embryos showed detectable PKH26-labelled fluorescent cells in the gonadal region. The relatively low frequency of gonadal localization may reflect several factors, including the presence of endogenous germ cells in recipients and the phylogenetic distance between the donor and recipient species, which may limit efficient colonization of the recipient gonadal niche. Based on the current results, one possible explanation is that the use of germ-cell-deficient recipients and more closely related recipient species may improve donor-cell colonization in future studies. Furthermore, PKH26 as a donor cell tracer exhibits significant limitations—such as dye dilution effects, transfer artifacts, fluorescence persistence, and nonspecific labeling—all of which may complicate data interpretation. However, this study primarily focuses on establishing an *in vivo* transplantation platform to investigate whether cultured cells possess germ cell characteristics. Consequently, future work should incorporate donor-specific genetic markers (e.g., transgenic fluorescent labels or species-specific molecular tags) for cell transplantation experiments. Overall, the transplantation experiment demonstrated that PKH26-labelled cultured cells remained detectable in recipient embryos for up to one week, with labelled cells observed in the gonadal region of a small subset of recipients. However, donor-specific molecular confirmation and evidence of functional germline contribution are required to determine whether these cells retain SSC activity after prolonged culture.

*In vitro* germ-cell differentiation provides a potential approach for producing gametes without continuous maintenance of mature broodstock and may therefore complement conventional breeding and surrogate-reproduction strategies. Teleost spermatogenesis generally proceeds through three major phases: mitotic proliferation and self-renewal of spermatogonia, meiotic differentiation into haploid spermatids, and spermiogenesis leading to mature spermatozoa [45,46]. Functional sperm derivation has been achieved in honmoroko (*Gnathopogon caerulescens*) from suspended testicular cells [47], while yellow river carp (*Cyprinus carpio haematopterus*) generated spermatid-like and sperm-like cells following retinoic acid (RA) treatment [48]. Similarly, southern catfish (*Silurus meridionalis*) SSCs underwent spermatogenic differentiation following combined RA and 11-ketotestosterone (11-KT) treatment [49]. In the present study, we adapted the hormonal induction protocol developed Zhang et al. [20] for the four-eyed sleepers (*Bostrychus sinensis*) to induce RnSSCs. Because the original protocol was developed using freshly isolated testicular cells, whereas the RnSSCs used here had undergone prolonged *in vitro* culture, differences in cellular state and culture microenvironment may have influenced their response to hormonal stimulation. Following adaptation of the previously reported induction conditions, cells bearing flagellum-like structures appeared by day 14, and sperm-like cells with condensed heads and elongated flagella were observed by day 16. Notably, this study demonstrated that even in the absence of testicular somatic cell feeder layers, hormonal stimulation could still trigger the expression of meiosis-related genes. This response may reflect hormone sensitivity retained by a subset of cells within the cultured population; however, the cellular basis of this response remains to be determined. Similar results were observed in Yellow River carp [48], and southern catfish [49]. In addition, hormone treatment also induced certain morphological alterations in a subset of cells, which may originate from non-germ cell differentiation. Given the heterogeneous nature of the cell population in this system, not all cells followed the spermatogenesis-like differentiation pathway. Therefore, we will subsequently employ limiting dilution purification to obtain homogeneous germ stem cell populations for induction experiments, enabling more precise analysis of germ-cell-specific differentiation responses. RT-PCR analysis further showed that the meiotic-associated markers *rec8* and *dmc1* were detected in hormone-treated RnSSCs but not in untreated controls [50,51], supporting the activation of meiotic-related genes under the induction conditions. The transcriptional activation of meiosis-related genes does not necessarily equate to the successful initiation and completion of meiosis. Cells may initiate the transcription of meiosis-related genes but could become arrested at early meiotic stages, failing to proceed with subsequent chromosomal events. Therefore, further validation through chromosomal analysis, ploidy detection, and functional assays is required to confirm the progression of meiosis. Moreover, since the current induction system yielded only a small proportion of cells exhibiting sperm-like morphology, we plan to optimize *in vitro* culture conditions or transplant germ-cells into closely related recipient species to obtain functional sperm in subsequent experiments.

## 5. Conclusions

Spermatogonial stem cells (SSCs), which possess self-renewal and gametogenic potential, represent a valuable cellular resource for fish reproductive research. In this study, we established a long-term cultured germ-cell population exhibiting characteristics consistent with an undifferentiated spermatogonial phenotype, with the expected diploid chromosome number (2*n* = 50) and stable expression of both germ-cell-associated and stem-cell-associated markers. Following transplantation into zebrafish blastulae, PKH26-labeled cells remained detectable in developing embryos, including in the gonadal region of a small subset of recipients. Following hormonal treatment, a subset of cells developed morphology consistent with spermatogenic differentiation, accompanied by detection of the meiosis-associated markers *rec8* and *dmc1*. The cultured cell population provides a potential renewable resource for further studies of germ-cell biology, differentiation, and transplantation in *R. nasutus*. However, functional SSC activity, germline contribution, and completion of meiosis were not demonstrated and require further validation.

## Figures and Tables

**Table 1:** Primer sequences used for RT-PCR.

Gene Name	Primer Sequence		Product Size (bp)
	Forward Primer	Reverse Primer	
*piwi*	CTTGCAAAACCGTGGTCAGG	GGGCTCCTTGTGAATGCTCT	562
*vasa*	CCTCATGTTCAGTGCCACCT	ACTTTGACCAGAGAGCGAGC	546
*nanog*	CCGCTCTGACCAACCTCAAT	TGTGGGTAGCTGGGGTAGAA	562
*oct4*	CCGACTCCCTCTGCATCATC	GACTCCAGAGCAGAACGGAC	506
*dmrt1*	AGTGCCAGAAATGCCGACTA	CACACTCCAGCCTGTTCTCA	600
*rec8*	GTCACACTGGCTGCTTCTCT	GGTAACTCTGCCCCACCAAA	583
*dmc1*	TTGGAGGAGGTGTGGAGAGT	TGGGCTTTTTGGGATCTGCT	528
*β-actin*	TCCGTGACATCAAGGAGAAGC	AACAAGAAGGATGGCTGGAAC	177

## Data Availability

All the data are available within the article.

## Supplementary Materials

The following supporting information can be downloaded at: https://www.mdpi.com/article/10.3390/biology15191779/s1, Figure S1. The spermatogonial cells of *R. nasutus* cultured for 7, 15, 60, 90, and 120 days, respectively. n: nucleus; ni: nucleolus. Bars = 100 μm; Figure S2. RT-PCR analysis of germ-cell-associated markers, stem-cell-associated markers, and somatic-cell-associated markers in RnSSC-like cells and gonadal tissues. Lanes from left to right: RnSSC-like cells, testis, ovary. Figure S3. Hormone-induced differentiation of RnSSC-like cells into distinct somatic cell. (A) Neuronal cells. (B) Flattened morphology cells. (C) Epithelial cells. (D) Astrocytes. Bars = 20 μm. Figure S4. RT-PCR detection of meiotic-associated markers in hormone-induced RnSSC-like cells, control RnSSC-like cells, and gonadal tissue. Lanes from left to right: hormone-induced RnSSC-like cells, control RnSSC-like cells, testis, ovary.
